# Comparative Metabolomics Study Revealed Difference in Central Carbon Metabolism between Sika Deer and Red Deer Antler

**DOI:** 10.1155/2020/7192896

**Published:** 2020-08-25

**Authors:** Hang Su, Chonghui Yang, Chenrong Jin, He Zhang, Chengcheng Yin, Yang Yang, Haoyuan Chen, Li Jing, Bin Qi, Daqing Zhao, Xueyuan Bai, Li Liu

**Affiliations:** ^1^Practice Innovations Center, Changchun University of Chinese Medicine, Changchun 130117, China; ^2^Jilin Ginseng Academy, Changchun University of Chinese Medicine, Changchun 130117, China; ^3^College of Pharmacy, Changchun University of Chinese Medicine, Changchun 130117, China; ^4^Research Center of Traditional Chinese Medicine, College of Traditional Chinese Medicine, Changchun University of Chinese Medicine, Changchun 130117, China

## Abstract

The antler regeneration has been well studied for the past two decades and adopted in the regenerative medicine model for studying on developmental biology. Despite our growing knowledge of functional molecules regulating antler regeneration, we still do not know whether antler from different deer species possess the exact same mechanism or not. Our previous comparative study between sika deer and red deer suggests that the metabolic pathways between them are profoundly different based on protein level. Therefore, the metabolomic technology is used to identify and quantify the metabolites in antler samples, providing interesting insights into differential metabolite profile of antlers between sika deer and red deer. The distinct metabolic characteristics of sika deer compared to red deer provide an opportunity to explain why the red deer antler with a larger size. The enrichment analysis of differential metabolites showed that three pathways including glycine and serine metabolism, methionine metabolism, and pterine biosynthesis had a significant difference between two antler groups.

## 1. Introduction

Deer antler, as the only mammalian regenerative organ, is a traditional Chinese medicine. The characterization of antler regeneration attracted ancient scholars of traditional Chinese medicine to develop it for the treatment of bone defect. The therapeutic effect of deer antler was documented in “Shennong's Classic of Materia Medica”, which is the earliest book of materia medica written more than two thousand years ago. It is the first record of “regenerative medicine” in the field of traditional Chinese medicine. The antler only harvested from sika deer and red deer was widely used in traditional Chinese medicine, even though other deer species also can produce antler. And comparative studies between sika deer antler and red deer antler play a vital role in whether traditional medicine or regenerative medicine study model. The antlers from both sika deer and red deer initiate regeneration from February to April [1]. The fast regeneration stage of their antlers is from May to July [2]. Following into autumn, the antler gradually loses velvet and vessels, resulting in calcification [3]. The weight of red deer antler will reach to 7 to 9 kg at late regeneration stage, whereas the sika deer antler with only 3 to 6 kg is considerably less than red deer [4]. We need more information about the differences of metabolites to interpret the weight difference between sika deer and red deer. The functional molecules regulating antler regeneration induced large interest for regenerative medicine researchers over the past decades. The antler generation through modified endochondral ossification was firstly reported by Banks and Newbrey [5]. The pedicle periosteum was verified to drive antler to regeneration through antler histogenesis and tissue depletion [6–8]. In addition to pedicle periosteum study, the antler growth center with the fastest growth rate is also regarded as the ideal model of regenerative medicine study [9–11].

The global transcriptomics analysis of sika deer antler growth center revealed that Wnt signaling also participated in chondrogenesis and osteogenesis during antler regeneration stage [12]. Molnár et al. identified two expression clusters for 36 genes that were mainly expressed in consecutive tissue zones of antler growth center of red deer [13]. The first gene expression cluster was found to involve with ribosome pathway, and the second cluster could be related with tumor biology. The pathways related to antler regeneration are similar but not exactly the same. Our previous study showed that the metabolic pathway between red deer antler and sika deer antler was profoundly different based on protein level [14]. The metabolic study was a promising technology for systematic research in antler metabolome. To precisely detect the metabolites related with tumor biology, targeted metabolomics workflow using the MRM technique for high-throughput profiling of metabolites in central carbon metabolism. The triple quadrupole mass spectrometry (QqQ-MS)-based multiple reaction monitoring (MRM) technique is widely used in targeted metabolomics because of high sensitivity and good reproducibility [15]. The data acquisition of known metabolites also significantly reduces the efforts on data processing and metabolite identification. The data process and metabolites identification were simplified through data acquisition based on reference metabolites.

The aim of our study was to investigate the antler metabolite profiles from sika deer and red deer based on high-throughput targeted metabolomics. We were able to analyze 200 polar metabolites related with central carbon metabolism and finally have detected metabolites for comparison between sika deer and red deer. A previous study that disclosed the key genes and proteins during the antler regeneration using transcriptomics and proteomics technologies suggested that antler regeneration was strongly associated with tumor biology. And the alteration of central carbon metabolism is a characteristic feature in tumor biology [16, 17]. Thus, the metabolomics targeted on central carbon metabolism will accurately work out essentially changed metabolic pathways of antler regeneration between red deer and sika deer.

## 2. Materials and Methods

### 2.1. Tissue Sampling

We randomly choose six male sika deer (SD1) and six male red deer (RD2) as study subjects in Chinese local deer farm, which was located in Shuangyang district, Changchun city, Jilin province (43°33′56.03^″^N, 125°29′23.15^″^E). All procedures of the sample collection were approved by the Animal Ethics Committee of Changchun University of Chinese Medicine. They are all 5-year-old deers with antler regeneration for 80 days after casting previous antlers. Antler tissues were harvested from anaesthetized deer. Antler growth center was obtained according Li's protocol [18]. And then, the antler growth center was sectioned sagittally by the electric saw to collect 5 mm to 8 mm thick tissue section. And then, tissue section was cut into small pieces, flash frozen, and ground into powder in liquid nitrogen and stored at -80°C ready for metabolomic study.

### 2.2. Metabolites Extraction Process

The prepared antler samples (50 mg) were extracted with a MeOH : ACN : H2O (1 : 1 : 1, *v*/*v*) solvent mixture. The mixture was vortexed and then frozen in liquid nitrogen. The samples were then allowed to thaw at room temperature and sonicated for 30 min. The freeze-thaw cycle was repeated three times. The samples were incubated at -20°C for 1 h to precipitate proteins, followed by 20 min centrifugation at 14,000 rcf and 4°C. The solvent of the resulting supernatant was evaporated to obtain semisolid in a vacuum dryer. And then, the extracts were reconstituted in 200 *μ*L of ACN : H2O (1 : 1, *v*/*v*), sonicated for 10 min and centrifuged 15 min at 14,000 rcf and 4°C to obtain the supernatants. The supernatants, stored at -80°C, were ready for LC/MS analysis.

### 2.3. Liquid Chromatography-Tandem Triple Quadrupole Mass Spectrometry

The LC-MS analysis was performed using the UPLC system (Agilent 1290) coupled to a triple quadruple mass spectrometer (Agilent 6460) in the multiple reaction monitoring (MRM) mode. And metabolites were monitored in both ESI positive and negative modes. Amide column (ACQUITY UPLC BEH, l.7 *μ*m, 2.1 × 100 mm) was selected to separate the metabolite sample. The column temperature was kept at 40°C. The flow rate was set to 0.3 mL/min, and the injection volume was 2 *μ*L. The mobile phase A was 25 mM ammonium acetate and 25 mM ammonium hydroxide in water, and B was acetonitrile. The gradient elution method was set as follows: 0-1 min: 95% B, 1-14 min: 95% B to 65% B, 14-16 min: 65% B to 40% B, 16-18 min: 40% B, 18-18.1 min: 40% B to 95% B, 18.1-23 min: 95% B. The parameters of ESI source: sheath gas temperature of 350°C, dry gas temperature of 350°C, sheath gas flow of 11 L/min, dry gas flow of 10 L/min, capillary voltage of 4000 V or -3500 V in positive or negative modes, nozzle voltage of 500 V, and nebulizer pressure of 30 psi. The dwell time for acquiring MRM transition was 3 ms. The total cycle time for MRM assay was 1.263 s. The raw data of MRM assay was analyzed by MRMAnalyzer to obtain area integration of peaks from each target metabolites [15, 19]. Detected metabolites in pooled samples with coefficient of variation (CV) less than 30% were regarded as reproducible measurements.

### 2.4. Data Analysis

The statistical analyses between two antler groups were conducted by evaluating the fold change and *P* value of metabolites. Welch *t*-test with unequal variances was used to determine the *P* value. Metabolites with the *P* value less than 0.05, fold-changes greater than 1.5, were considered as the significantly changed metabolites between two groups. The differential antler metabolites between sika deer and red deer were analyzed by MetaboAnalyst 4.0 software for metabolic pathway analysis, which is based on database source including HMDB, and KEGG [20].

## 3. Results

### 3.1. Identified Metabolites from Sika Deer Antler and Red Deer Antler

To identify different metabolite profiles between sika deer and red deer antler, targeted high-throughput metabolomics was conducted by LC-MS coupled with multiple reaction monitoring (MRM) analysis. And 113 metabolites were identified in deer antler metabolome based on targeted metabolomics method. For each of detected metabolite, the quantitative and qualitative information was output as an Excel file (Table S1). The metabolite intensity, fold change, and *P* value between two antler groups were used to quantify, while the qualitative information is composed of metabolite name, KEGG ID, and HMDB ID. To reveal the metabolomic difference between two antler groups, 37 of 113 metabolites were found to change significantly in abundance with 2.0-fold change (*P* < 0.05). Compared with red deer, 25 metabolites among significantly changed metabolites were upregulated in sika deer antler, and 12 metabolites upregulated in red deer.

### 3.2. Differential Metabolic Pathways between Sika Deer Antler and Red Deer Antler

**Figure 1:**
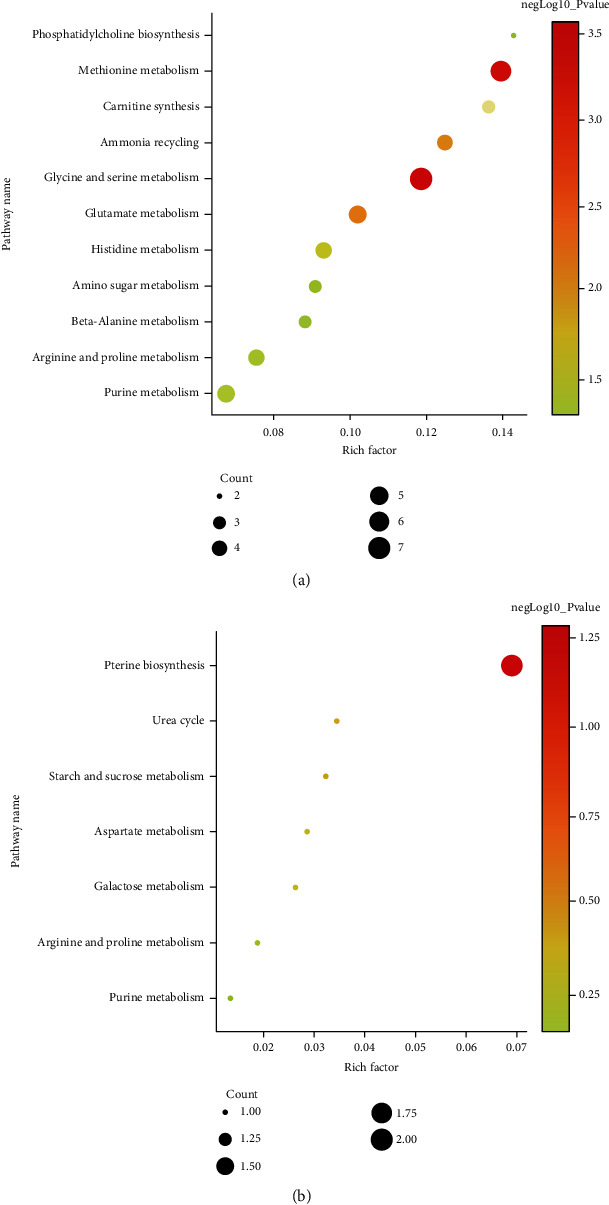
Metabolic pathway enrichment analysis for significantly changed metabolites between sika deer and red deer. (a). Pathways involved in upregulated metabolites in sika deer. (b). Pathways involved in upregulated metabolites in red deer.

The upregulated 25 metabolites and downregulated 12 metabolites in sika deer were further proceeded for pathway enrichment analysis using MetaboAnalyst, respectively [20]. The enrichment analysis showed that two pathways including glycine and serine metabolism and methionine metabolism had a significant difference between two antler groups (Table 1, Figure 1).

Of 25 upregulated metabolites in sika deer, 7 metabolites were enriched into glycine and serine metabolism, and 6 metabolites were involved in methionine metabolism (Table 1). Serine, methionine, vitamin B6, and S-adenosylhomocysteine were significantly upregulated in sika deer antler (Table 2).

Among glycine and serine metabolism pathway, four metabolites were highly abundant in sika deer (Table 3).

Of 12 upregulated metabolites in red deer, biopterin, neopterin, and sepiapterin were involved in pterine biosynthesis pathway (Table 4).

## 4. Discussion

### 4.1. L-Methionine and Glutathione Metabolism

As a key intermediate metabolite involved in central carbon metabolism, methionine was transformed to S-adenosylmethionine (SAM) by methionine adenosyltransferase (MAT) in methionine cycle [21]. And then, the demethylation of SAM generated S-adenosylhomocysteine (SAH) under the methyltransferase reaction. The content of methionine can determine the ratio of SAM to SAH, which impact the DNA methylation reactions. The content of methionine and SAH in sika deer antler was higher than that of red deer. This result indicated that DNA methylation level was higher in sika deer antler compared to red deer antler [22]. A previous study demonstrated that DNA methylation regulated the antler regeneration through changing the methylation of regeneration-associated genes [23]. The difference of DNA methylation level between sika deer and red deer antler may impact the size of antler. With the lower DNA methylation level, red deer will possess the larger size antler. Notable, the further study was necessary to verify this viewpoint.

Besides the regulation of DNA methylation, methionine plays an important role in the production of glutathione (GSH) through cystathionine pathway [24]. S-adenosylhomocysteine (SAH), by-product of methyltransferase reactions, was hydrolyzed to homocysteine (Hcy). Accompany with vitamin B6, Hcy reacted with serine to obtain cystathionine through cystathionine *β*-synthase (CBS). The cleavage of cystathionine releases free cysteine for GSH synthesis under *γ*-cystathionase. Serine, methionine, vitamin B6, and S-adenosylhomocysteine were upregulated in sika deer antler metabolome (Table 2), and it indicated that the higher abundance of GSH may be produced to resist oxidative stress in sika deer during antler regeneration stage. Under the same feeding operation, sika deer face more serious oxidative stress to maintain the growth of antler compared with red deer. It also explains why the sika deer have the small size antler based on oxidative stress.

### 4.2. Glycine and Serine Metabolism

As the important source of folate biosynthesis, seine and glycine are essential to regulate the folate-mediated one-carbon metabolism, which is responsible for purine synthesis and the remethylation of homocysteine to methionine [25, 26]. And folate metabolism pathway also participated in nucleotides synthesis for the assemble of DNA and RNA [26]. This methylation reaction regenerates tetrahydrofolate (THF) from 5-methyltetrahydrofolate (methyl-THF) [25]. Purine nucleotide bases are also synthesized from the folate metabolism pathway through the intermediate 10-formyltetrahydrofolate, which is produced from 5,10-methylene-THF under methylenetetrahydrofolate dehydrogenase 1 (MTHFD1) [25]. Folate metabolism pathway in sika deer was stimulated to maintain folate homeostasis under the high abundance of glycine, serine, and purine (Table 3). Previous study had shown that the folate deficiency and hyper homocysteine had a detrimental impact on osteoblasts, osteoclast in regard to bone formation and remodeling [27]. To avoid the accumulation of homocysteine in stimulated folate metabolism pathway of sika deer, betaine and homocysteine were catalyzed to produce dimethylglycine and methionine by betaine-homocysteine S-methyltransferase in a pathway that is folate independent. This may explain why methionine levels are higher in sika deer antler as compared to red deer (Table 2). The high level of S-adenosylhomocysteine in sika deer may lead to slow antler growth rate compared with red deer because of detrimental impact on bone remodeling [28]. In addition, glycine, proline, and hydroxyproline consist of more than 50% of total amino acids (AAs) in collagen, which helps the regeneration of cartilage [29, 30]. The higher levels of glycine and proline (Table 3) will enhance the cartilage regeneration in sika deer antler in part to offset the effects of high level of homocysteine.

### 4.3. Pterine Biosynthesis

In the pterine biosynthesis, 7,8-dihydroneopterin, which originates from guanosine triphosphate (GTP), is a precursor of neopterin and tetrahydrobiopterin (BH4) [31, 32]. Besides de novo synthesis from GTP, BH4 can be synthesized through salvage pathway [31]. Sepiapterin, through the salvage pathway, can be reduced to regenerate BH4 by sepiapterin reductase in the case of BH4 deficiencies [33]. The high abundance of sepiapterin and sepiapterin reductase in red deer indicated that the biosynthesis of BH4 depended on sepiapterin salvage pathway (Table 4) [14]. BH4 is the key cofactor of nitric oxide synthase (NOS), which involved in the catalysis of arginine to nitric oxide [34, 35]. Beier et al. reported that NOS played a vital role in chondrocyte proliferation during endochondral ossification and loss of NOS promoted prehypertrophic chondrocyte differentiation during cartilage development [36]. Thus, it is necessary for antler development to maintain the BH4 level in red deer. The activation of BH4 salvage pathway in red deer inhibited the ROS production during regeneration stage [37]. It may be the key factor that determined the larger size of red deer compared to sika deer because of the activation of BH4 salvage pathway [33]. We also found that the content of oxidized biopterin in red deer antler is much higher than sika deer. It is conceivable that BH4 may act as the main antioxidant to resist oxidative stress in red deer (Table 4). Consequently, the antioxidation ability during antler regeneration stage is significantly different between sika deer and red deer. Metabolomic study further showed that the relative abundance of neopterin was specifically higher in red deer antler (Table 4). During the endochondral ossification stage, neopterin is produced in osteo macrophages at the expense of BH4 under the immune activation [38–40]. A positive correlation between levels of neopterin with macrophage was observed in endochondral ossification [39, 40]. The decrease in macrophages would affect endochondral ossification, which led to a delayed hard callus formation [40]. The high abundance of neopterin is a key factor for red deer to keep the high efficiency of endochondral ossification that regulates the antler regeneration.

## 5. Conclusions

In this study, we use multiple reaction monitoring (MRM)-based targeted metabolomics to determine the antler metabolomic profiling. With large numbers of MRM transitions acquired with 12 deer antler samples, then, we used MRMAnalyzer to process MRM dataset automatically without any manual intervention for saving data analysis time. Finally, we identified 113 metabolites involved with central carbon metabolism. Among these identified metabolites, there are 25 abundant metabolites in sika deer antler and 12 abundant metabolites in red deer antler. The enrichment analysis of differential metabolites indicated that glycine and serine metabolism, methionine metabolism, and pterine biosynthesis were distinctly different metabolic pathways between sika deer antler and red deer antler during antler regeneration stage. We firstly profiled the comparative metabolome between sika deer and red deer antlers, both of which were treated as the authentic source of antler velvet in the “Chinese Pharmacopoeia”. The comparative metabolomics also provides insights into the potential chemo-marker related to identifying different antler species. Notably, this high throughput metabolomic method is appropriate to discover the metabolomic variations in deer antlers at the temporal and spatial scales in the future study.

## Figures and Tables

**Table 1:** Enrichment analysis for significant changed metabolites.

Pathway	Metabolites	*P* value	FDR
Glycine and serine metabolism	Glycine; glyceric acid; L-glutamic acid; L-serine; sarcosine; L-methionine; S-adenosylhomocysteine	0.000313	0.0184

Methionine metabolism	Glycine; L-serine; sarcosine; L-methionine; L-homoserine; S-adenosylhomocysteine	0.000375	0.0184

**Table 2:** Significantly changed metabolites involved L-methionine and glutathione metabolism.

HMDB ID	Metabolite name	RD2/SD1	*P* value
HMDB00187	L-Serine	0.466	3.47E-10
HMDB00696	L-Methionine	0.461	3.46E-08
HMDB01545	Vitamin B6	0.704	0.00729
HMDB00939	*S*-Adenosylhomocysteine	0.201401502	2.12E-09

**Table 3:** Significantly changed metabolites involved glycine and serine metabolism.

HMDB ID	Metabolite name	RD2/SD1	*P* value
HMDB00123	Glycine	0.336	2.19E-10
HMDB00187	L-Serine	0.466	3.47E-10
HMDB01366	Purine	0.565	1.44E-05
HMDB00162	L-Proline	0.834	7.78E-08

**Table 4:** Significantly changed metabolites involved pterine biosynthesis.

HMDB ID	Metabolite name	RD2/SD1	*P* value
HMDB00845	Neopterin	8.107	9.63E-04
HMDB00238	Sepiapterin	2.523	5.01E-07

## Data Availability

The data used to support the findings of this study are included within the supplementary information files.

## Supplementary Materials

Supplementary Materials Table S1: the metabolites identified in sika deer and red deer antler.
